# Bridging from Brain to Tumor Imaging: (*S*)-(−)- and (*R*)-(+)-[^18^F]Fluspidine for Investigation of Sigma-1 Receptors in Tumor-Bearing Mice †

**DOI:** 10.3390/molecules23030702

**Published:** 2018-03-20

**Authors:** Mathias Kranz, Ralf Bergmann, Torsten Kniess, Birgit Belter, Christin Neuber, Zhengxin Cai, Gang Deng, Steffen Fischer, Jiangbing Zhou, Yiyun Huang, Peter Brust, Winnie Deuther-Conrad, Jens Pietzsch

**Affiliations:** 1 Department of Neuroradiopharmaceuticals, Institute of Radiopharmaceutical Cancer Research, Helmholtz-Zentrum Dresden-Rossendorf, 04318 Leipzig, Germany; s.fischer@hzdr.de (S.F.); p.brust@hzdr.de (P.B.); w.deuther-conrad@hzdr.de (W.D.-C.); 2 Department of Diagnostic Radiology, PET Center, Yale University School of Medicine, New Haven, CT 06519, USA; zhengxin.cai@yale.edu (Z.C.); henry.huang@yale.edu (Y.H.); 3 Department of Radiopharmaceutical and Chemical Biology, Institute of Radiopharmaceutical Cancer Research, Helmholtz-Zentrum Dresden-Rossendorf, 01328 Dresden, Germany; r.bergmann@hzdr.de (R.B.); t.kniess@hzdr.de (T.K.); b.belter@hzdr.de (B.B.); c.neuber@hzdr.de (C.N.); j.pietzsch@hzdr.de (J.P.); 4 Department of Neurosurgery and Biomedical Engineering, Yale University School of Medicine, New Haven, CT 06519, USA; gang.deng@yale.edu (G.D.); jiangbing.zhou@yale.edu (J.Z.); 5 Technische Universität Dresden, School of Science, Faculty of Chemistry and Food Chemistry, 01062 Dresden, Germany

**Keywords:** [^18^F]fluspidine, carcinoma, glioblastoma, melanoma, sigma-1 receptor, dedicated small animal PET/CT

## Abstract

Sigma-1 receptors (Sig1R) are highly expressed in various human cancer cells and hence imaging of this target with positron emission tomography (PET) can contribute to a better understanding of tumor pathophysiology and support the development of antineoplastic drugs. Two Sig1R-specific radiolabeled enantiomers (*S*)-(−)- and (*R*)-(+)-[^18^F]fluspidine were investigated in several tumor cell lines including melanoma, squamous cell/epidermoid carcinoma, prostate carcinoma, and glioblastoma. Dynamic PET scans were performed in mice to investigate the suitability of both radiotracers for tumor imaging. The Sig1R expression in the respective tumors was confirmed by Western blot. Rather low radiotracer uptake was found in heterotopically (subcutaneously) implanted tumors. Therefore, a brain tumor model (U87-MG) with orthotopic implantation was chosen to investigate the suitability of the two Sig1R radiotracers for brain tumor imaging. High tumor uptake as well as a favorable tumor-to-background ratio was found. These results suggest that Sig1R PET imaging of brain tumors with [^18^F]fluspidine could be possible. Further studies with this tumor model will be performed to confirm specific binding and the integrity of the blood-brain barrier (BBB).

## 1. Introduction

In clinical oncology, major efforts are dedicated to the optimization of cancer treatment either with chemotherapeutics or radiation therapy [1]. For detection, diagnostic prognosis, and follow-up after treatment of tumors positron emission tomography (PET) is a widely applied tool able to image tumor-specific biochemical processes in vivo with radioactive probes [2]. One of the most widely applied radiotracers for that purpose is the glucose analog [^18^F]fluorodeoxyglucose ([^18^F]FDG) which accumulates in tissues with high metabolic activity [2,3]. However, it has shown some limitations in the detection of tumors like prostatic carcinoma DU145 [4] or PC-3 xenografts [5], A431 xenografts [6], FaDu tumors [7], A375 tumors [8], and in U87-MG glioblastoma [9,10]. Hence, not all tumor species show high glucose uptake (non-FDG-avid tumors) and consequently might be overlooked in a tumor screening study [11,12,13,14]. Furthermore, for brain tumors, the high background uptake of glucose in healthy brain makes the detection of small or low-grade tumors almost impossible [15]. Therefore, radiolabeled probes based on small molecules, amino acids, peptides, or antibodies targeting different biomarkers, e.g., tumor metabolism, cellular proliferation, or hypoxia were developed for the visualization and characterization of brain tumors with PET [16].

One promising approach for the use of PET radiotracers in oncology is the development of sigma receptor (SigR) ligands [17,18,19]. There is evidence that both SigR subtypes, sigma-1 receptors (Sig1R) and sigma-2 receptors (Sig2R), play important roles in cancer biology [17]. Studies with different human and rodent tumor cell lines have proven that there exists a high density of SigR binding sites in cancer [20]. While the Sig2R are regarded as potential markers for cellular proliferation [19,21,22], the stress-activated Sig1R are associated with the endoplasmic reticulum interface and are involved in the regulation of calcium signaling [23]. Furthermore, Aydar et al. has described a link between Sig1R expression and the tumor aggressiveness [24], suggesting that this receptor subtype may be a potential marker for the diagnosis and prognosis of (brain) tumors [23]. Hence, imaging of Sig1R with PET might contribute to a better understanding of the tumor physiology, the pathophysiological function of Sig1R, and the development of antineoplastic drugs [25].

Several PET radiotracers have been developed for imaging of Sig1R, such as [^18^F]1-3-fluoropropyl-4-((4-cyanophenoxy)-methyl)piperidine ([^18^F]FPS) [26], 1-(4-[^18^F]fluorobenzyl)-4-[(tetrahydrofuran-2-yl)methyl]piperazine [27], [^11^C]1-(3,4-dimethoxyphenethyl)-4-(3-phenylpropyl)piperazine ([^11^C]SA4503) [28], [^18^F]6-(3-fluoropropyl)-3-(2-(azepan-1-yl)ethyl)benzo[d]thiazol-2(3H)-one ([^18^F]FTC-146) [29] and the ^18^F-labeled 1,4-dioxa-8-azaspiro[4.5]decane derivative from our group [30]. Recently, we developed another two promising radioligands (*S*)-(−)-[^18^F]fluspidine and (*R*)-(+)-[^18^F]fluspidine [31,32], which both have been successfully used to image Sig1R in healthy mice [31] and piglets [33]. Furthermore, (*S*)-(−)-[^18^F]fluspidine was applied to humans in a first-in-man study [34]. The two enantiomers differ in their affinity towards Sig1R ((*R*)-(+)-fluspidine: *K*_i_ = 0.57 nM; (*S)*-(−)-fluspidine: *K*_i_ = 2.3 nM), which is suggested to influence their different pharmacokinetics [33]. The *S*-enantiomer, showing fast and reversible binding kinetics in brain, was chosen for the first-in-man evaluation [34]. Furthermore, a phase 1 clinical trial (drug occupancy study, ClinicalTrials.gov identifier: NCT03019289) is currently underway to investigate (*S*)-(−)-[^18^F]fluspidine for imaging and quantification of Sig1R in brain with pharmacokinetic modeling. 

The current study was performed to investigate both enantiomers of [^18^F]fluspidine for their ability to image the Sig1R availability in tumors. Starting from the cellular level, we investigated the Sig1R synthesis with Western blot and the accumulation of (*S*)-(−)- and (*R*)-(+)-[^18^F]fluspidine in the human prostatic cancer cells DU145 and PC3, as well as in the cell lines A431 and FaDu (squamous carcinoma), A375 (malignant melanoma), and U87-MG (glioblastoma). Supported by the promising in vitro results, PET scans with (*S*)-(−)-[^18^F]fluspidine were performed in mice bearing five different heterotopically (peripheral) implanted tumors, representing both FDG-avid and non-FDG-avid cancers. Furthermore, an orthotopic brain tumor model in mice was chosen and used in PET scans to investigate the applicability of the two radiotracers with special regard to brain tumors (pilot study). 

## 2. Results

### 2.1. Cell Specific Expression/Synthesis of Sig1R

**Figure 1:**
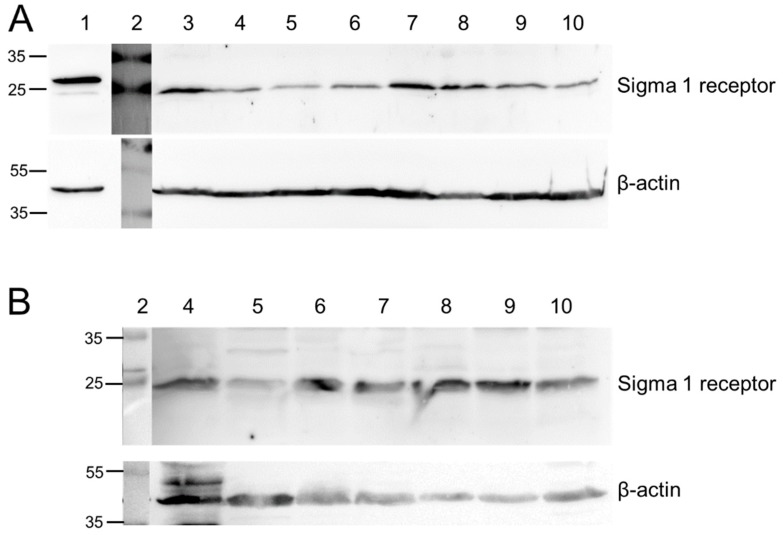
Detection of the Sig1R protein in (**A**) cell lysates and (**B**) tumor lysates. (1) HEK-S1R (Sig1R-overexpressing, transgenic cells, positive control), (2) protein standard, (3) HEK, (4) FaDu, (5) PC3, (6) DU145, (7) A431, (8) A375, (9) U87-MG, (10) NCI-H292. Expected band at 25 kDa. β-actin was used as loading control.

The Sig1R expression was analyzed in lysates from melanoma (A375), squamous cell/epidermoid carcinoma (FaDu, A431), prostate carcinoma (DU145, PC3), glioblastoma (U87-MG), and lung carcinoma (NCI-H292) cells grown in vitro in cell culture as well as in mouse heterotopic tumor. As demonstrated in Figure 1, we detected the Sig1R protein in all cell lines as well as in the explanted tumor xenografts.

### 2.2. Cellular Accumulation of [^18^F]fluspidine

**Figure 2:**
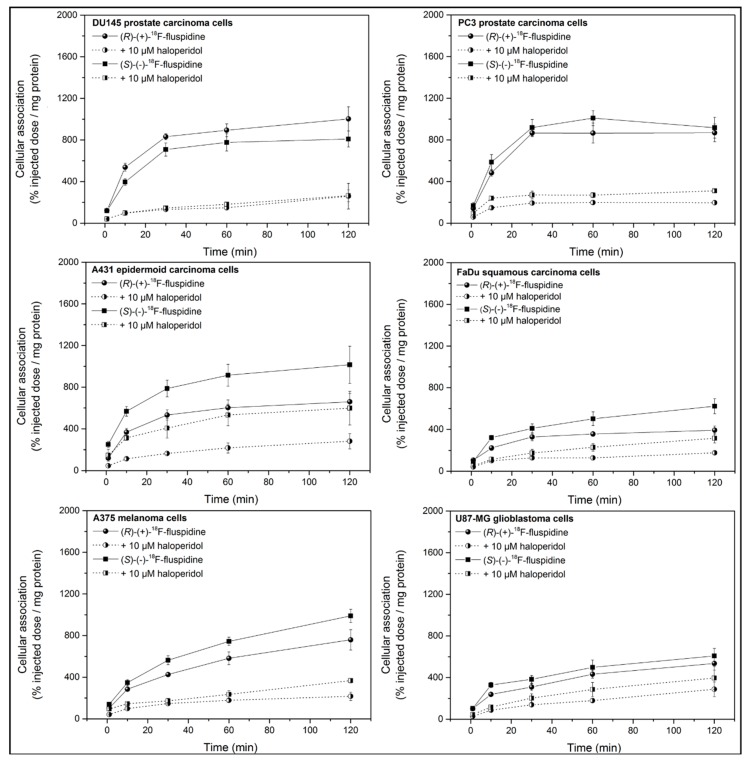
Cellular accumulation of (*S*)-(−)- and (*R*)-(+)-[^18^F]fluspidine in the human tumor cells DU145, PC3, A431, FaDu, A375, and U87-MG in vitro. Blocking experiments were performed by preincubation (for 10 min) with 10 µM haloperidol. Results are given as percentage of injected dose (%ID) per mg protein (mean ± SD; *n* ≥ 8 for (*R*)-(+)-[^18^F]fluspidine and *n* = 7 for (*S*)-(−)-[^18^F]fluspidine).

Six human tumor cell lines were used to study the cellular accumulation (Figure 2) of (*S*)-(−)-[^18^F]fluspidine and (*R*)-(+)-[^18^F]fluspidine in vitro following a published protocol [30].

Substantial cellular accumulation of both (*S*)-(−)- and (*R*)-(+)-[^18^F]fluspidine was observed in all investigated cancer cell lines. The extent of the cellular accumulation at 120 min differs between the cell lines and the highest cellular accumulation of (*S*)-(−)-[^18^F]fluspidine was observed for A431 and A375 cells, with levels slightly higher than those of (*R*)-(+)-[^18^F]fluspidine. Furthermore, high cellular accumulation of both radiotracers was observed in the two prostate carcinoma cell lines and the lowest values were obtained with FaDu and U87-MG cells.

Blocking studies were performed using 10 µM haloperidol which significantly reduced the cellular accumulation of both (*S*)-(−)- and (*R*)-(+)-[^18^F]fluspidine in the cell lines with an inhibitory effect of 35–77%. These data demonstrate specific binding of both enantiomers of [^18^F]fluspidine to Sig1R in all cell lines under investigation. 

### 2.3. Small Animal PET Imaging

**Figure 3:**
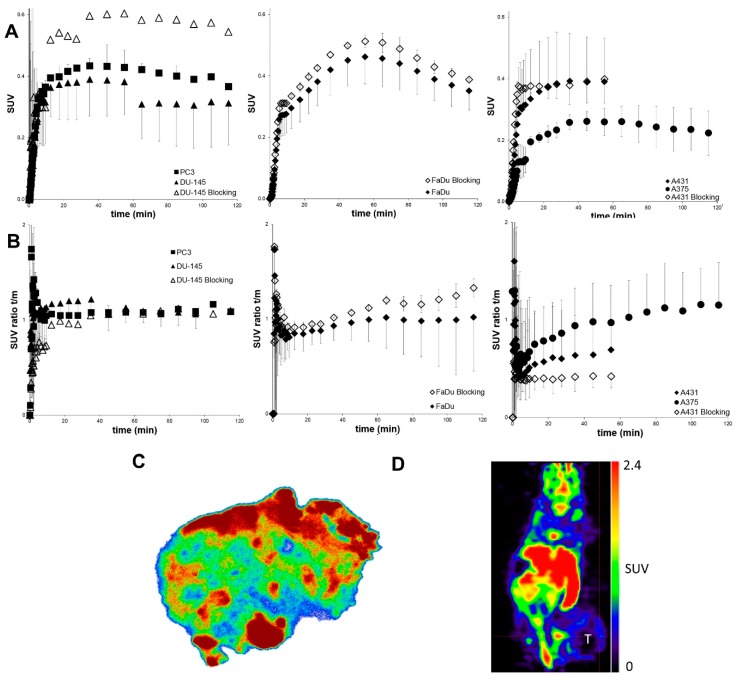
Small animal PET imaging in mice bearing different heterotopic tumors (*n* = 2/tumor model; *n* = 1 for DU145 blocking) after i.v. injection of (*S*)-(−)-[^18^F]fluspidine. (**A**) The maximal tumor uptake corresponds to standardized uptake values (SUV) of 0.25 and 0.6 while a blocking effect is not visible (mean SUV ± SD). (**B**) Tumor-to-muscle SUV ratios of the respective animals (mean SUV ratio ± SD). (**C**) In vitro Sig1R autoradiography with (*S*)-(−)-[^18^F]fluspidine of an explanted U87-MG tumor, grown heterotopically in a mouse, with heterogeneous activity distribution. (**D**) Representative coronal PET image of a FaDu tumor bearing mouse at 50–60 min p.i. (tumor highlighted, T).

Small animal PET was performed in tumor-bearing mice after i.v. injection of (*S*)-(−)-[^18^F]fluspidine. The results obtained for the five heterotopically (peripheral) implanted tumors in terms of standardized uptake values (SUV) or the tumor-to-muscle ratio are presented in Figure 3. Although there is uptake of (*S*)-(−)-[^18^F]fluspidine in all investigated tumor models and the suitability of this radioligand for detection of Sig1R in tumors was indicated by in vitro autoradiography in an explanted heterotopic U87-MG tumor (Figure 3C), the extent of accumulation is low. The highest accumulation of the radiotracer was observed in FaDu (squamous cell carcinoma, *n* = 2) and PC3 (human prostate cancer, *n* = 2) tumors. Furthermore, the tumor-to-muscle ratio revealed a low signal from the surrounding tissue (Figure 3D). Administration of haloperidol did not significantly reduce the tracer uptake in the respective tumor models. Hence, no specific binding of (*S*)-(−)-[^18^F]fluspidine to Sig1R was found in the heterotopically implanted tumors.

**Figure 4:**
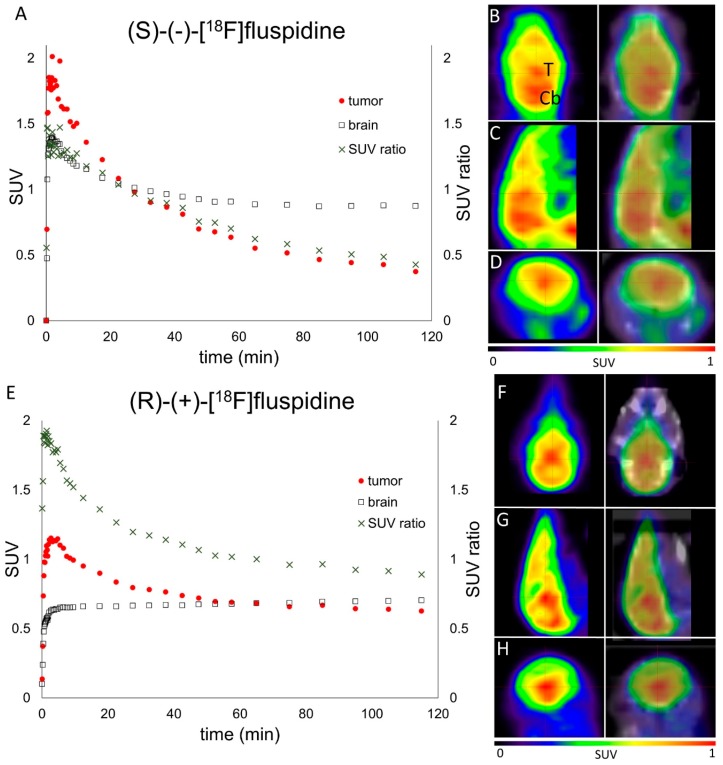
In vivo PET/CT imaging of mice with orthotopically implanted glioblastoma cells (U87-MG) after i.v. administration of (*S*)-(−)-[^18^F]fluspidine (*n* = 2) (**A**–**D**) or (*R*)-(+)-[^18^F]fluspidine (*n* = 3) (**E**–**H**). (**A**,**E**) Higher SUV of the tumor compared to the whole brain up to 25 or 50 min p.i., (*S*)-(−)-[^18^F]fluspidine or (*R*)-(+)-[^18^F]fluspidine, resulting in tumor-to-background SUV ratios >1 and hence tumor visibility at early time points. (**B**–**D**,**F**–**H**) Summed PET and PET/CT frames from 3–15 min p.i. in coronal (**B**,**F**), sagittal (**C**,**G**) and transaxial (**D**,**H**) views (T-tumor, Cb-cerebellum).

Subsequently, a pilot study using small animal PET/CT after orthotopic tumor cell implantation (U87-MG) into the brain revealed high tumor uptake and a favorable tumor-to-background ratio (Figure 4A,E) following the administration of (*S*)-(−)-[^18^F]fluspidine (*n* = 2) or (*R*)-(+)-[^18^F]fluspidine (*n* = 3), respectively. The PET/CT images (Figure 4B–D,F–H) showed a clear separation of tumor from the brain. However, further PET/MR and ex vivo studies need to be performed to confirm specific binding of the ligands in the tumor.

## 3. Discussion

Our group has recently developed (*R*)-(+)- and (*S*)-(−)-[^18^F]fluspidine [31], two specific radioligands for Sig1R PET with promising preclinical properties and distinctive kinetics [33,35]. In this publication, we investigated the suitability of both enantiomers of [^18^F]fluspidine to image Sig1R in different mouse tumor models. Current investigations [30] show strong expression of Sig1R and correlation with pathologic tumor tissue in human esophageal squamous cell carcinoma [36], prostate cancer [37,38], myeloma [39], melanoma [20,40], and glioma [41]. To assess the expression of the Sig1R protein in different human tumors, cell lines from epidermoid carcinoma, melanoma, and glioblastoma were investigated with Western blot in the current study. The results showed a positive signal for all investigated tumor cell lines, which represent both FDG-avid- and non-FDG-avid cancer entities, and confirmed substantial Sig1R expression in these tumor types.

Next, we investigated the accumulation and specific binding of (*S*)-(−)- and (*R*)-(+)-[^18^F]fluspidine in the selected cancer cell lines in order to evaluate the potential of both radiotracers to address specific oncological questions. In general, all the cell lines showed accumulation of both enantiomers of [^18^F]fluspidine and treatment with haloperidol significantly reduced the radiotracer binding [42,43]. Although haloperidol shows affinity to other receptors (i.e., Sig2R and dopamine D_2_), it was used as blocking agent in the current study as the binding of [^18^F]fluspidine is highly Sig1R selective [31]. Furthermore, 1′-benzyl-3-methoxy-3H-spiro[[2]benzofuran-1,4′-piperidine, the lead compound of the ligand development resulting in [^18^F]fluspidine, did not show any relevant binding towards more than 60 neurotransmitter receptors, ion channels, and neurotransmitter transporters [44,45]. Hence, results from the blocking study confirm Sig1R specific binding of (*S*)-(−)- and (*R*)-(+)-[^18^F]fluspidine in the cancer cell lines.

Given the limitations of [^18^F]FDG for tumor imaging, we performed in vivo investigations using small animal dynamic PET to examine the tracer uptake and specific binding and therefore the suitability of (*S*)-(−)-[^18^F]fluspidine as tumor imaging agent. Although we could show in vitro the expression of Sig1R in heterotopically grown U87-MG tumor by autoradiography with (*S*)-(−)-[^18^F]fluspidine, the analysis of the PET scans performed in human tumor xenograft models revealed comparatively low accumulation of this radioligand in the respective tumors. It is reflected in particular by low tumor-to-muscle ratios due to low specific signal in tumor, i.e., binding displaceable by haloperidol. This is due to areas of necrosis in these tumor models where radiotracer accumulation is not expected [46]. Another possible reason is that interstitial oncotic pressure generated by proliferating cancer cells might have reduced the tumor blood flow and prohibited tracer uptake [47]. Furthermore, the lack of specific uptake for [^18^F]fluspidine in heterotopic (peripheral) tumors [48,49] may be caused by differences in the tumor microenvironment and vascularization in these models [50,51]. Additionally, as shown by Figure 3C, the heterogeneity of the tumor tissue results in different levels of radiotracer uptake and applying ROI analysis to the whole tumor region results in low mean SUV. 

In a subsequent pilot study we performed PET imaging with [^18^F]fluspidine in orthotopically implanted brain tumors. In general, [^18^F]fluspidine has been shown to be suitable for brain imaging [31,33,52] and thus, we hypothesize that these radioligands are suitable for imaging of Sig1R expressing brain tumors. Preliminary data from these experiments revealed a tumor-to-background SUV ratio (TBR) of >1 for early time points and thus visualization of the tumor in the mouse brain appeared to be feasible. However, at this point of investigation, without having the fraction of specific binding of both enantiomers proven with suitable experiments (e.g., pre-blocking with haloperidol or SA4503) a comparison of [^18^F]fluspidine with established radiotracers is not possible. Further studies with this orthotopic brain tumor model are ongoing to test the BBB integrity, to confirm the specific binding and to further evaluate both enantiomers of [^18^F]fluspidine as brain tumor imaging agents.

## 4. Materials and Methods 

### 4.1. Radiochemistry

Enantiomerically pure (*S*)-(−)-[^18^F]fluspidine and (*R*)-(+)-[^18^F]fluspidine were prepared on a TRACERlab FX_N_ synthesizer (GE Healthcare, Waukesha, WI, USA) as described in previous publications [33]. The radiochemical purity of (*R*)-(+)- or (*S*)-(−)-[^18^F]fluspidine was >99%, and the molar activity at the end of the synthesis was 69.2 ± 35.8 GBq/µmol (*n* = 9) and 56.6 ± 17.3 GBq/μmol (*n* = 7), respectively.

### 4.2. Cell Culture

Human malignant melanoma line A375 (ATCC CRL-1619), human squamous cell/epidermoid carcinoma cell lines FaDu (ATCC HTB-43) and A431 (ATCC CRL-1555), androgen-independent human malignant prostate adenocarcinoma lines PC3 (ATCC CRL-1435) and DU145 (ATCC HTB-81), and human likely glioblastoma cell line U87-MG (ATCC HTB-14) were used. Cells were routinely cultivated in Dulbecco’s modified Eagle’s medium (A375, A431, PC3, U87-MG), Eagle’s Minimum Essential Medium (DU145) or RPMI 1640 medium (FaDu) supplemented with 10% (*v*/*v*) heat-inactivated fetal calf serum (FCS), penicillin (100 U/mL), streptomycin (100 µg/mL), glutamine (4 mM), 1% HEPES (1 M; A431 and U87-MG cells only) at 37 °C and 5% CO_2_ in a humidified incubator. Cells were passaged twice a week by mild enzymatic dissociation using 0.25% trypsin/EDTA [30,53].

### 4.3. Immunoblotting (Western Blot)

Tumor samples were processed as described earlier [54]. Preparation of cell lysates, SDS-PAGE and immunoblotting were performed as described elsewhere [55]. In brief, 40–80 µg protein per lane was transferred to PVDF membranes using a semi-dry transfer system (Bio-Rad Laboratories, Hercules, CA, USA). Membranes were blocked for 60–90 min with non-fat dry milk powder (5%, *w*/*v*) in Tris-buffered saline containing 0.05% (*v*/*v*) Tween 20 (TBS-T). For detection of Sig1R, membranes were incubated with primary antibodies in bovine serum albumin (BSA, 2% *w*/*v*) in TBS-T (PA5-30372, 1:500, Thermo Fisher Scientific (Waltham, MA, USA), (tumor samples), respectively, ab53852, 1:200, Abcam (Cambridge, UK) (cell lysates)), followed by incubation with peroxidase-conjugated secondary antibody (anti-rabbit IgG, A0545, 1:5000, Sigma-Aldrich, Steinheim, Germany). Proteins were visualized using Super Signal West Pico/Femto Chemiluminescent Substrate (Thermo Fisher Scientific) and a CELVIN^®^S Chemiluminescence Imaging system (Biostep, Burkhardtsdorf, Germany). For detection of loading control, membranes were stripped and further processed using mouse anti-β-actin antibody (A5316, 1:1000, Sigma-Aldrich) and anti-mouse IgG (A9044, 1:10,000, Sigma-Aldrich) as described elsewhere [54].

### 4.4. Cellular Accumulation

Radiotracer uptake studies with (*S*)-(−)-[^18^F]fluspidine/(*R*)-(+)-[^18^F]fluspidine (stock solution 1.50–1.75 MBq/mL; molar activity at application time: 69 GBq/µmol for (*R*)-(+)-[^18^F]fluspidine and 56 GBq/µmol for (*S*)-(−)-[^18^F]fluspidine) were performed in monolayer cultures. Therefore, the cells were seeded in 24-well plates at a density of 1.0 × 10^5^ cells/mL and grown to confluence. The tracer cell uptake experiments were performed in quadruplicate in PBS at 37 °C for 1, 10, 30, 60, and 120 min using an activity of 0.3–0.5 MBq/well for each tracer in a total volume of 500 µL (independent experiments with and without blocking, 2–3 for (*R*)-(+)-[^18^F]fluspidine and 2 for (*S*)-(−)-[^18^F]fluspidine). For blocking experiments, the cells were pre-incubated for 10 min with 10 µM of haloperidol (100 µL). After the tracer uptake was stopped with 1 mL ice-cold PBS, the monolayer cells were washed three times with PBS and dissolved in 0.5 mL NaOH (0.1 M containing 1% sodium dodecylsulfate, *w*/*v*). The radioactivity in cell extracts was then measured with a Cobra II gamma counter (Canberra-Packard, Meriden, CT, USA) and decay-corrected. Activity measurements were corrected for nonspecific tracer binding determined in empty (cell-free) plates using the same experimental conditions. Total protein concentration in cell extracts was determined by the bicinchoninic acid assay (BCA; Pierce, Rockford, IL, USA) using bovine serum albumin as protein standard. Uptake data for all experiments are expressed as percentage of injected dose per mg protein (%ID/mg protein). 

### 4.5. Heterotopic Tumor Model and Small Animal PET Imaging

Animal experiments were carried out according to the guidelines of the German Regulations for Animal Welfare. The protocol was approved by the local Ethical Committee for Animal Experiments (reference numbers 24D-9168.11-4/2007-2 and 24-9168.21-4/2004-1).

For the generation of subcutaneous tumors, DU145, PC3, A431, FaDu, A375, and U87-MG cells were used and cultivated as described elsewhere [30,54]. Tumor cells were harvested, washed in PBS, and transferred to 0.9% sodium chloride solution (5 × 10^6^ cells/100 µL). Nine weeks old male (prostate cancer cells) and female (other cancer cells) NMRI Foxn1^nu/nu^ mice (weight: 35.9 ± 4.6 g) were purchased from Janvier Labs or from the specific pathogen-free breeding facility of the Experimental Centre of the Faculty of Medicine Carl Gustav Carus, Technische Universität Dresden. General anesthesia of mice was induced with inhalation of desflurane 12% (*v*/*v*) (Suprane, Baxter, Unterschleißheim, Germany) in 40% oxygen/air (gas flow 0.5 L/min), and was maintained with desflurane 8% (*v*/*v*). The single-cell suspension (100 µL) was subcutaneously injected into the right hind leg of mice [56]. Tumor size was monitored trice a week by caliper measurements and tumor volume was calculated. The animals were visually inspected daily. Tumor-bearing mice entered imaging studies 18–24 days past tumor cell injection, when tumor size reached a volume of about 400 to 700 mm^3^. 

For PET investigations, anesthesia was performed as described above. In the PET experiments, 7.6 ± 3.0 MBq of (*S*)-(−)-[^18^F]fluspidine or (*R*)-(+)-[^18^F]fluspidine was administered intravenously over 1 min into a tail vein. Dynamic PET imaging was performed for up to 2 h with a dedicated small animal tomograph (microPET P4, Siemens Medical Solutions, Knoxville, TN, USA). Data acquisition was performed in 3D list mode. A transmission scan was carried out prior to the injection of the radiotracer using a ^57^Co point source. The list mode data were sorted into sinograms using a framing scheme of 12 × 10 s, 6 × 30 s, 5 × 300 s and 9 × 600 s frames. The frames were reconstructed by Ordered Subset Expectation Maximization applied to 3D sinograms (OSEM3D) with 14 subsets, 6 OSEM3D iterations, 2 maximum a posteriori (MAP) iterations, and 0.05 beta-value for smoothing and corrected for attenuation. The pixel size was 0.8 by 0.8 by 1.2 mm, and the resolution in the center of field of view was 1.85 mm. The reconstructed data were converted into ECAT7 format and processed using the ROVER software (ABX GmbH, Radeberg, Germany). Summed frames from 30 to 60 min post injection (p.i.) were used to define the regions of interest (ROI). The ROI were located over the tumor and the muscle of the contralateral hindleg and the results expressed as SUV or SUV_tumor/muscle_ ratio.

### 4.6. Orthotopic Brain Tumor Model: Stereotactic Intracranial Tumor Cell Inoculation and PET/CT Imaging

All procedures were approved by the Institutional Animal Care and Utilization Committee (IACUC) of Yale University. The procedures for cell culture are described in detail elsewhere [57]. Mice were purchased from Charles River Laboratories. Intracranial U87-MG-luc mouse xenografts were established in 5–6 week-old female athymic nude mice (Crl:NU(NCr)-Foxn1^nu^). The animals were anesthetized via intraperitoneal injection of a ketamine/xylazine mixture. After positioning in a stereotactic apparatus, a skin incision was made until the bregma was visible, a 0.45 mm hole was drilled into the skull at 2 mm lateral and 0.5 mm posterior to the bregma and a 30 G needle attached to a 10 μL Hamilton syringe was inserted 3 mm deep into the brain tissue. The needle remained for 2 min at this position followed by the injection of 50,000 U87-MG cells in 5 μL of PBS (2.5 μL/min) into the right striatum with an UltraMicroPump (UMP3, World Precision Instruments, Sarasota, FL, USA). After the whole volume was injected, the needle was kept in place for another 2 min and then quickly withdrawn from the brain. The hole was closed with bone wax (Ethicon Inc., Somerville, NJ, USA) and the incision site sutured. Finally, the mice were inspected after narcosis until they were fully awake. For the three consecutive days after implantation the animals received s.c. injections of Meloxicam (5 mg/kg) for pain relief and anti-inflammatory treatment. The tumor growth was monitored weekly by bioluminescence imaging (IVIS Spectrum, PerkinElmer, Waltham, MA, USA). PET/CT experiments were performed 4 weeks after tumor cell inoculation using the Inveon PET/CT scanner (Siemens Medical Solutions, Knoxville, TN, USA). Dynamic PET scans were acquired for 2 h following i.v. injection of 3.5 MBq ± 2.6 of (*S*)-(−)-[^18^F]fluspidine or (*R*)-(+)-[^18^F]fluspidine. The list mode data were sorted into sinograms using a framing scheme of 12 × 10 s, 6 × 30 s, 5 × 300 s and 9 × 600 s frames. The frames were reconstructed by OSEM3D, corrected for attenuation based on the CT data resulting in a pixel size of 0.78 by 0.78 by 0.8 mm and a resolution in the center of the FOV of 1.64 mm. ROIs were defined with ROVER for whole brain and the tumor region and the results expressed as standardized uptake value.

## 5. Conclusions

In this study we investigated the feasibility for tumor imaging of two Sig1R specific radiolabeled enantiomers of [^18^F]fluspidine using different mouse tumor models, with special regard to brain tumor imaging. The results support the use of the two radiotracers as imaging agents for clinical oncological applications. Both enantiomers of [^18^F]fluspidine showed promising results in the cellular accumulation experiments and encouraged us to further investigate these radiotracers in different tumor models. Although the heterotopic models are not suitable for PET imaging with [^18^F]fluspidine, the results obtained with an orthotopic brain tumor model support the use of (*S*)-(−)-[^18^F]fluspidine and (*R*)-(+)-[^18^F]fluspidine for brain tumor imaging. However, further studies with this orthotopic brain tumor model will be needed to investigate the integrity of the blood-brain barrier, to confirm the specific binding to Sig1R and to investigate the suitability of [^18^F]fluspidine for brain tumor imaging.
